# Effectiveness of Injury Prevention Programs on Developing Quadriceps and Hamstrings Strength of Young Male Professional Soccer Players

**DOI:** 10.2478/hukin-2013-0074

**Published:** 2013-12-31

**Authors:** Abdolhamid Daneshjoo, Nader Rahnama, Abdul Halim Mokhtar, Ashril Yusof

**Affiliations:** 1 Faculty of Physical Education and Sport Science, Shahid Bahonar University of Kerman, Kerman, Iran.; 2 Faculty of Physical Education and Sport Science, University of Isfahan, Isfahan, Iran.; 3 Faculty of Medicine, University of Malaya, Kuala Lumpur, Malaysia.; 3 Sports Centre, University of Malaya, Kuala Lumpur, Malaysia.

**Keywords:** knee, strength, professional soccer player, the 11+, HarmoKnee

## Abstract

Muscular strength is an important factor which is crucial for performance and injury prevention in most sports. The purpose of this study was to evaluate the effects of the FIFA’s Medical Assessment and Research Centre 11+ and HarmoKnee injury prevention programs on knee strength of young professional male soccer players. Thirty-six soccer players (age: 18.9 ± 1.4 years) were divided equally into three groups; the 11+, HarmoKnee and control groups. The programs were performed for 24 sessions. Hamstring and quadriceps strength was measured using the Biodex System 3 at 30°, 60° and 90° of knee flexion. The 11+ increased quadriceps strength in the dominant leg by 19.7% and 47.8% at 60°and 90° knee flexion, respectively, and in the non-dominant leg by 16%, 35.3% and 78.1 % at 30°, 60° and 90° knee flexion, respectively. The HarmoKnee group, however, showed increased quadriceps strength only at 90° i.e., by 85.7% in the dominant leg and 73.8% in the non-dominant leg. As for hamstring strength, only the 11+ group demonstrated an increment by 24.8% and 19.8% at 30° and 60° knee flexion in the dominant leg, and in the non-dominant leg, by 28.7% and 13.7% at 30° and 60° knee flexion, respectively. In conclusion, both warm-up programs improve quadriceps strength. The 11+ demonstrated improvement in hamstring strength while the HarmoKnee program did not indicate any improvement. We suggest adding eccentric hamstring components such as Nordic hamstring exercise to the HarmoKnee program in order to enhance hamstring strength.

## Introduction

Risk factors of overuse injuries are generally categorized into intrinsic and extrinsic (Bahr and Holme, 2003; Fousekis et al., 2011). Off the intrinsic factors, muscle strength is one of the modifiable factors. Furthermore, it is an important key for efficient motor performance during activities of daily living (Lu et al., 2012), and a main factor of physical performance such as playing soccer (Lehance et al., 2009). Poor muscle strength has been also suggested as a factor predisposing an athlete to injury (Mjølsnes et al., 2004). Moreover, bilateral strength differences between the dominant and non-dominant leg especially in lower body reportedly can lead to improper control of body movement and consequently injury (Knapik et al., 1991; Schiltz et al., 2009; Daneshjoo et al., 2013). The bilateral strength imbalance plays a critical role in sports with asymmetric kinetic patterns like soccer (Tourny-Chollet et al., 2002; Daneshjoo et al., 2013).

Measurement of muscle strength plays an important role in the evaluation and prediction of muscular condition in addition to functional capacity. Moreover, it is resourceful in monitoring changes quantitatively and efficacy of an intervention or training program (Lu et al., 2012). Additionally, muscular strength is also crucial in injury prevention through dynamic joint stabilization (Holcomb et al., 2007; Park et al., 2010). The quadriceps acts as a dynamic stabilizer of the knee joint, whereas the hamstrings mainly protect against anterior subluxation through the action of dynamic protagonists on the anterior cruciate ligament (Park et al., 2010).

Previous studies have reported that playing soccer presents a higher risk of injuries compared with other team sports (Engebretsen et al., 2008; Fousekis et al., 2011). Due to the nature of the sport, most injuries in soccer are localized in the lower extremities (almost 70%), and the knee being the most common site with having 54% of the injuries (Junge et al., 2004; Kiani et al., 2010). Knee injuries are shown to pose a serious hazard to athletes, at times causing lengthy absence from competition and imposing enormous costs on teams and players (Rahnama et al., 2009). To address the issue of prevention of knee injuries among soccer players, data on modifiable risk factors such as strength should be widely studied (Bahr and Holme, 2003; Brito et al., 2010).

Based on literature, there are two commonly used comprehensive injury prevention programs by professional soccer players. FIFA’s Medical Assessment and Research Centre (F-MARC) has developed the 11+ injury prevention program. The 11+ program has been shown to successfully reduce the prevalence of knee injuries in female soccer players (Soligard et al., 2008). Another related study investigated the effects of a 10 week 11+ program on isokinetic strength of young non-professional male soccer players (Brito et al., 2010). The study reported a significant increase in quadriceps strength in the dominant leg alone, while the hamstrings strength increased in both legs (Brito et al., 2010). Meanwhile, the HarmoKnee injury prevention program which was introduced by Kiani et al. (2010) showed a reduction of 77% in knee injury incidences among soccer players. Importantly, both the 11+ and HarmoKnee prevention programs were designed to be soccer-specific which could easily be included into regular warm-up exercise sessions at no additional cost and equipment. To our knowledge, studies that investigated the effect of the 11+ and HarmoKnee prevention programs on strength are scarce. Therefore, with respect to injury prevention among professional players, the main aim of this study was to investigate the effects of eight weeks of the 11+ and HarmoKnee programs on isometric strength of young professional male soccer players.

## Material and Methods

### Participants

Thirty-six young male professional soccer players were studied (mean ± SD; age: 18.9 ± 1.4 years, body mass: 73.6 ± 6.3 kg; body height: 181.3 ± 5.5 cm). Subjects with at least five years of experience playing soccer at a professional level with regular training and without any history of major lower limb injuries or diseases participated in this study. All participants provided written informed consent prior to the commencement of the study. The research was approved by the ethical committee of the Institute of Research Management and Monitoring, University of Malaya and the Sports Centre Research Committee.

### Procedure

At the mid- season of 2011, the coaches and team managers from three professional teams were invited to a four-hour instructional course which aimed to introduce the intervention programs without revealing much detail as to the types of exercises as well as the specific aims of the study. Three under-21 (U21) teams from professional soccer clubs volunteered to participate in this study. They were picked randomly and matched using knee strength. One-way ANOVA did not show significant difference in pre-test between the 11+, HarmoKnee and control groups at all knee angles of the quadriceps and hamstrings (p>0.05).

All groups attended a workshop separately to discuss the prescribed training program. They also received video instructions and illustrations on the exercises prior to the intervention. All of the training sessions were supervised by one of the researchers at any given time, to ensure their compliance with the programs. The soccer players were instructed on how to perform the exercises correctly. Verbal encouragements were given throughout the training period to help subjects concentrate on the quality of their movements. The subjects were then familiarized with the isokinetic machine and the isokinetic system for a knee extension and flexion protocol. The settings were recorded to ensure the same positioning for all experimental tests. The programs started on the 15^th^ of April, 2011, and were completed on the 15^th^ of June, 2011 (24 sessions).

### The warm-up injury prevention programs

#### The 11+ program

This program consists of three parts (27 exercises). The initial part includes running exercises combined with active stretching (part one). It is followed by six different sets of exercises to develop strength, balance, muscle control and core stability (part two). The final part is composed of running exercises combined with soccer-specific exercises (part three). The different levels of difficulty would improve the program’s efficiency and enable players to individually adapt to the program. Total program duration was 20 to 25 minutes (Table 1). The intervention program was carried out three times per week as a warm-up program before starting regular practice.

#### The HarmoKnee program

HarmoKnee injury prevention program includes five parts. The program begins with warm-up exercises at low speeds, followed by muscle activation, balance, strength, and ends with core stability components. The program takes approximately 20–25 minutes to be completed (Kiani et al., 2010). Similar to the 11+, the HarmoKnee was also performed three times per week as a warm-up before starting regular practice (Table 2).

#### Control group

The control group was asked to carry on with their regular warm-up and training throughout the study period. In addition, before commencement of the study, the control group was assured that they would receive the intervention program in the subsequent season.

### Isometric test

Strength of quadriceps and hamstring in both legs was measured using a Biodex Isokinetic Dynamometer (Biodex 3, 20 Ramsay Rode, Shirley, New York, USA). The Biodex System 3 has been shown to be a reliable instrument for collecting net peak torque (NPT) data (Drouin et al., 2004). Before each testing session, the dynamometer was calibrated in accordance with the manufacturer’s recommendations. Participants performed a general cardiovascular warm-up for at least five minutes on a Monark cycle ergometer at a moderate pace (60 RPM), which was then followed by 10 minutes of dynamic stretching (such as walking lunges, squats, and heel-toe-walks) concentrated on lower limbs (Rahnama et al., 2005).

The subject was seated on a chair while the upper body was stabilized with straps secured across the shoulders, chest and hips. The cuff of the dynamometer’s lever arm was attached proximal to the malleoli of the ankle. Dynamometer orientation was fixed at 90° and tilted at 0°, while the seat orientation was fixed at 90° and the seatback tilted at 70°–85°. The rotational axis of the knee joint was aligned with the dynamometer’s rotational axis. The seating position of each of the subjects was recorded carefully and repeated during the post-test. Subject positioning and device set-up were based on Biodex System 3 manufacturer’s guidelines, and similar to the ones which may be found in literature (Brito et al., 2010; Iga et al., 2009). The isometric quadriceps and hamstring torques were measured at 30°, 60° and 90° of knee flexion. These knee flexions are commonly used to evaluate the isometric strength at all ranges of motion in the knee joint (Parulytė et al., 2011; Steffen et al., 2008). The players performed 5 s maximal contractions at each knee flexion angle. Between two contractions at the same angle, the players had a 10 s pause while they were given a 20 s rest between contractions at different angles. The order of testing was randomized for the dominant and non-dominant legs. Encouragement through verbal coaching and visual feedback was given to all subjects. Net peak torque (Nm) was taken as the maximum value achieved during the three contractions (Steffen et al., 2008). For assessment of hamstring and quadriceps strength, the tests were performed twice. The pre-testing was conducted one week prior to the intervention program and the post-test was recorded eight weeks after the pre-test (three days after the final training session). All tests were conducted in the same order for each player at pre- and post-tests, between 8 and 11 am (Rahnama et al., 2005; Steffen et al., 2008). Testing was performed by a member of the research team who was blinded to each subject’s intervention group.

### Statistical analysis

To compare the isometric strength between times (pre- and post-tests), groups (11+, HarmoKnee, control), target angles (30°, 60°, 90°), and legs (dominant, non-dominant), the 2×3×3×2 (time vs group vs angle vs leg) repeated measures mixed design ANOVA was used separately for quadriceps and hamstrings muscles as described by Holcomb et al. (2007). In case of statistical significance, the post-hoc Bonferroni test was conducted. The Levene’s test was employed for assessing homogeneity of variance among groups (p>0.05). Furthermore, the Kolmogorov-Smirnov test was employed for assessing normality of the distribution of scores (p>0.05). The effect sizes of each variable was tested using partial eta (η) squared (0.01=small effect, 0.06=medium effect, and 0.14=large effect). A significant level was accepted at 95% confidence level for all statistical parameters (p<0.05).

## Results

### NPT of the quadriceps muscle

**Figure 1:**
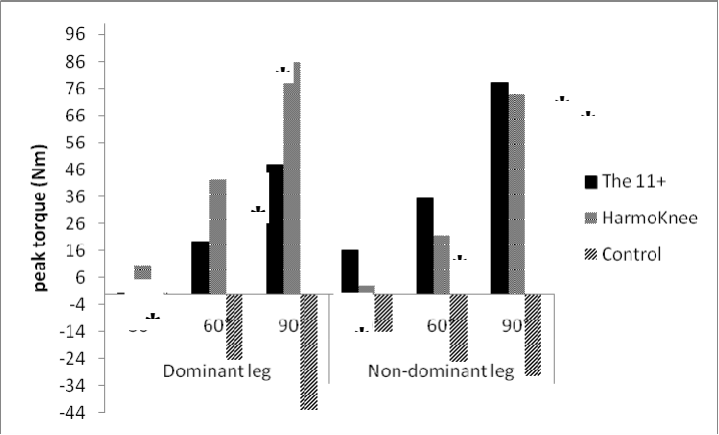
Percentage of change from pre-test to post-test in quadriceps strength (* p<0.05).

The means of quadriceps’ NPT in pre- and post-tests of the groups are presented in Table 3. The mixed ANOVA analysis showed a significant main effect between times (F_1,33_=6.39, p=0.016). The partial eta-squared statistic indicated a large effect size (0.16). There were significant interactions between time (pre- and post-tests) and knee flexion angles (30°, 60°, 90°) (F_2,32_=3.73, p=0.035) with a large effect size (0.19). The results showed significant interaction between time and group (F_2,32_= 9.178, p =0.001) with a large effect size (0.36). The Bonferroni post-hoc test in the 11+ group showed a significant increase of NPT in the dominant leg by 19.7% and 47.8% at 60°and 90° knee flexion, respectively; and in the non-dominant leg, NPT was increased by 16%, 35.3% and 78.1 % at 30°, 60°and 90° knee flexion, respectively. In the HarmoKnee group, NPT increased significantly (p<0.05) by 85.7% and 73.8% in both dominant and non-dominant legs, respectively, only at the 90° knee flexion. The results showed no significant difference in the control group (p>0.05) (Figure 1).

### Quadriceps NPT at different knee angles (30°, 60° and 90°)

The mixed ANOVA analysis showed significant main effect differences between angles (F_2,32_=379.11, p=0.000) with a large effect (0.96). The post-hoc test revealed that in the 11+group, there was a significant difference in the NPT of quadriceps muscles in both dominant (F_2,10_=7.291, p=0.011) and non-dominant legs (F_2,10_=10.981, p=0.003). The HarmoKnee group also showed a significant difference between the NPT of quadriceps’ muscles in the dominant (F_2,10_=7.954, p=0.009) and non-dominant legs (F_2,10_=6.591, p=0.015). The results did not show any significant differences in the control group (p>0.05).

### NPT of the hamstring muscles

**Figure 2:**
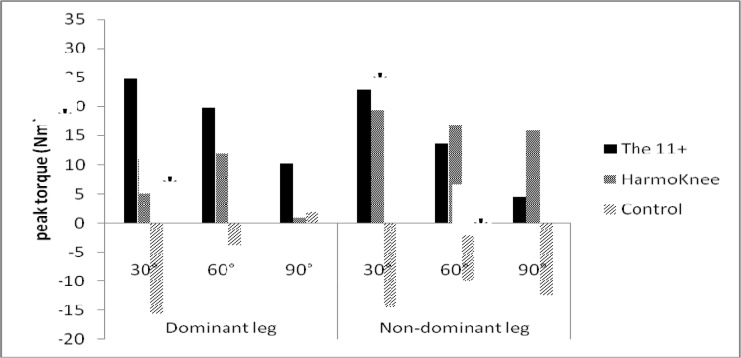
Percentage of change from pre-test to post-test in hamstring strength (* p<0.05).

The mean values of strength of the hamstring muscles in pre- and post-tests of the groups are presented in Table 4. The mixed ANOVA indicated no significant differences between times (F_1,33_=2.67, p=0.111). There were also no significant interactions between time and angle (p>0.05). However, the results showed significant interaction between time and group (F_2,33_=3.764, p=0.034). The partial eta squared statistics indicated a large effect size (0.19). In the 11+ group, hamstrings strength increased significantly (p<0.05) by 24.8% and 19.8% at 30° and 60° knee flexion in the dominant leg and by 28.7% and 13.7% at 30° and 60° in the non-dominant leg. The results indicated no significant differences in HarmoKnee and control groups (p>0.05) (Figure 2).

### Hamstrings NPT between knee angles (30°, 60° and 90°)

The mixed ANOVA analysis showed significant main effect differences between angles (F_2,32_=121.37, p=0.001) with a large effect size (0.88). Significant differences were shown only in the 11+ between the hamstring muscles of the non-dominant leg (F_2,10_=9.554, p=0.001). The results did not show any significant differences in HarmoKnee and control groups (p>0.05).

### Comparison of NPT between legs and groups

The mixed ANOVA analysis showed no significant difference in the quadriceps strength between the dominant and non-dominant legs (F_1,33_=0.509, p=0.481). Therefore, a significant difference was observed in the hamstrings between the legs (F_1,33_=21.345, p=0.001). The results showed no significant difference between groups in the quadriceps and hamstrings (p>0.05).

## Discussion

The aim of this study was to investigate the effect of the FIFA 11+ and HarmoKnee warm-up injury prevention programs on isometric strength in young professional male soccer players. One of the main findings of our study was an increase in quadriceps strength in the HarmoKnee group at 90° knee flexion by 85.7% and 73.8% in dominant and non-dominant legs, respectively. On the other hand, the 11+ program also showed increases in quadriceps strength in the dominant leg by 19.7% and 47.8% at 60° and 90° of knee flexion, respectively, and by 16%, 35.3% and 78.1% at 30°, 60° and 90° knee flexion, respectively, in the non-dominant leg. It seems that both programs have the potential of improving quadriceps’ strength. Related to this study, Brito and colleagues (2010) reported that the 11+ improved isokinetic NPT of the quadriceps at 60°.s^−1^ and 180°.s^−1^ in the dominant leg. Moreover, they observed that the 11+ increased hamstring NPT at 60°.s^−1^ in the dominant leg as well as at 60°.s^−1^ and 180°.s^−1^ in the non-dominant leg (Brito et al., 2010).

The general mechanisms that may have caused quadriceps’ net peak torque to improve in this study were an increase in body temperature, increasing the blood flow to the muscles, elasticity of the muscles and neuron activity (Sander et al., 2013) which is defined as an increase in muscle efficiency to produce force after a warm-up program (Sale, 2002), and possibly an increased rate of cross-bridge formation (Yamaguchi and Ishii, 2005). The 11+ and HarmoKnee programs are multifaceted and focus on core stability, balance, and neuromuscular control for soccer-specific skills that promote proper motion patterns (Kiani et al., 2010; Soligard et al., 2008). These programs also focus on body control (hip control and knee alignment) that prevents excessive knee valgus when playing soccer (Kiani et al., 2010; Soligard et al., 2008). Few studies have shown that, when these factors were incorporated into preventive programs, the rate of injuries was reduced (Kiani et al., 2010; Olsen et al., 2005; Soligard et al., 2008).

Maximal isometric quadriceps strength was found at 90° knee flexion in the dominant and non-dominant legs. Therefore, maximal isometric hamstring strength was found at 30° knee flexion in both legs. Muscle force basically depends on the amount of overlap between actin and myosin filaments in the sarcomere (length–force relation) (Sasaki and Ishii, 2005). It seems there are optimal overlaps between filaments in quadriceps and hamstring fibers at 90° and 30° knee flexions, respectively. In contrast with the hamstring fibres, quadriceps muscle fibres produce more contraction while the knee is in flexion due to optimum overlap between filaments. The present finding showed that 90° is the optimum flexion angle for measuring isometric quadriceps strength, while 30° is the optimum flexion angle for measuring isometric hamstring strength in young male professional soccer players.

Our results did not confirm any strength differences between legs in the quadriceps muscles. The present findings are in agreement with Ditroilo et al. (2010) and Stoll et al. (2000), who reported no significant differences in the quadriceps isometric strength between left and right legs in male athletes. Similarly to the present study, Rahnama et al. (2005) found no significant difference between the two legs in isokinetic NPT of the quadriceps in elite soccer players. Practically, the dominant leg is used to handle an object or to lead out, while the non-dominant leg has the main role of providing postural support. This definition of footedness is commonly accepted by researchers (Oshita and Yano, 2010; Daneshjoo et al., 2012). Professional soccer players can perform kicking of the ball bilaterally and prefer to use both legs in different situations. The quadriceps acts as prime movers to produce knee extension in kicking of the ball. This could be the possible cause of lack of quadriceps strength differences observed between dominant and non-dominant legs in professional soccer players. Conflicting results were reported by Schiltz et al. (2009) on male professional basketball players. They found significant differences in isokinetic quadriceps strength at 60°.s^−1^ (Schiltz et al., 2009). These contrasting results may be explained by the differences in the types of sport and strength tests used. In addition, specific demands such as neuromuscular control patterns during landing and cutting tasks are different between soccer and basketball (Cowley et al., 2006).

We found that the hamstrings strength increased significantly only in the 11+ group i.e. by 24.8% and 19.8% at 30° and 60° knee flexion in the dominant leg and by 28.7% and 13.7% at 30° and 60° knee flexion in the non-dominant leg. However, no significant differences were shown in HarmoKnee and control groups. Both intervention programs included elements which aimed to improve hamstring strength. In the 11+ it was an eccentric Nordic hamstring (it is a partner exercise where the subject attempts to resist a forward-falling motion using his hamstrings to maximize loading in the eccentric phase) while the HarmoKnee program included a concentric hamstring curl component (it is a partner exercise where the partner attempts to push your leg until your knee bend at 90° angle while you resist). Mjølsnes et al. (2004) compared the effect of a 10-week training program with two different exercises; hamstring curl and Nordic hamstrings among 21 male soccer players. They reported no changes in isometric hamstring strength at 30°, 60° and 90° knee flexion for the hamstring curl group, while the Nordic hamstring group showed a significant increase in all hamstring strength tests. Perhaps, the hamstring exercise in the HarmoKnee program is not sufficient to provide changes in hamstrings strength during the 8-week training period. Further studies including individual training components on quadriceps and hamstrings strength before and after intervention would help determine the effect of specific elements.

It was found that the hamstring muscle of the dominant leg was stronger than the non-dominant leg. This was also observed by Tourny-Chollet et al. (2002) who investigated 21 (22-year-old) amateur soccer players. They showed significant differences between isokinetic NPT of the hamstring muscles in the dominant and non-dominant legs. They concluded that the hamstrings of the dominant leg generally tend to be stronger than that of the non-dominant leg (Tourny-Chollet and Leroy, 2002). This pattern was explained by higher unilateral demands of hamstrings muscles in stabilizing actions in certain specific soccer skills such as landing and jumping (Cheung et al., 2012). In contrast, Rahnama et al. (2005) reported that hamstrings isokinetic strength in the non-dominant leg was more than that of the dominant leg in professional soccer players. These contradictory results may be attributed to the type of tests used to measure strength. In the present study, an isometric strength test was measured while Rahnama and colleagues (2005) used an isokinetic strength test.

Low hamstring strength is a risk factor for hamstring strains (Mjølsnes et al., 2004). In soccer, hamstring strains account for 12–17% of all injuries (Andersen et al., 2003). The likely explanation for this trend is that there are bilateral hamstring strength deficits between legs in soccer players. Bilateral strength imbalance has also been associated with injury (Rahnama et al., 2005). Schiltz et al. (2009) concluded that knee injuries among professional players were associated with bilateral strength and functional asymmetries. Knapik et al. (1991) revealed that athletes had a higher hamstring injury rate when the right hamstring was 15% stronger than the left hamstrings. Therefore, the results of our study indicated that the young professional soccer players are exposed to higher hamstring injury risks as well as impaired match-play performance.

## Conclusion

In conclusion, our results showed that both multifaceted, soccer-specific prevention programs which combine strength, neuromuscular control, balance and proper motion patterns without using special equipment can improve isometric quadriceps strength. We also found that the 11+ is relatively better at improving hamstring strength as compared to the HarmoKnee program. Maximal isometric quadriceps strength was found at 90° knee flexion while maximal isometric hamstring strength was found at 30° knee flexion. No significant difference was observed between legs in isometric strength of the quadriceps. In contrast, the hamstring muscle in the dominant leg was stronger than the non-dominant leg. It is suggested that the 11+ program could be implemented and incorporated into regular soccer practice as a warm-up program before starting technical and tactical drills. Further modifications of the HarmoKnee program may be required, for example by adding more training elements, especially Nordic hamstring exercise to fully realise the hamstring strength.

## Figures and Tables

**Table 1:** The 11+, exercises and duration of the structured warm-up program applied

**Exercise**	**Duration**

**Part 1: Running**	**8 minutes**
Straight ahead, hip out, hip in, circling partner, shoulder contact, quick forward & backwards (The course is made up of 6 to 10 pairs of parallel cones, approx. 5–6 m apart. 6 running items, each item 2 sets)	
**Part 2: Strength, Plyometric and Balance**	**10 minutes**
**The bench:** Static (Lift the body up while supporting on your forearms, pull your stomach in, and hold the position for 20–30 s), alternate legs (Lift each leg in turn, holding for a count of 2 s) and one leg lift and hold (Lift one leg about 10–15 cm off the ground, and hold the position for 20–30 s), (3 items, each item 3 sets)	
**Sideways bench:** Static (Lie on your side with the knee of your lowermost leg bent to 90 degrees, lift your uppermost leg and hips until your shoulder, hip and knee are in a straight line. Hold the position for 20–30 s), raise & lower hip (Lower your hip to the ground and raise it back up again. Repeat for 20–30 s), with leg lift (Lift your uppermost leg up and slowly lower it down again. Repeat for 20–30 s) (3 items, 3 sets on each side)	
**Hamstring:** Beginner (3–5 repetition, 1 set), intermediate (7–10 repetition, 1 set), advanced (12–15 repetition, 1 set). (Kneel on a soft surface. Lean forward as far as you can. When you can no longer hold the position, gently take your weight on your hands) (3 items)	
**Single-leg stance:** Hold the ball (Balance on one leg whilst holding the ball with both hands), throw the ball to a partner, test your partner (each of you in turn tries to push the other off balance in different directions), (3 items, each item 2 sets and each set 30 s)	
**Squats:** With toes raised (Perform squats by bending your hips and knees to 90 degrees), walking lunges (As you lunge, bend your leading leg until your hip and knee are flexed to 90 degrees), oneleg squats (3 items, each item 2 sets)	
**Jumping:** Vertical jumps, lateral jumps (Jump approx. 1m sideways from the supporting leg on to the free leg), box jumps (Alternate between jumping forwards and backwards, from side to side, and diagonally across the cross) (3 items, each item 2 sets)	
**Part 3: running exercise**	**2 minutes**
Across the pitch, bounding (Run with high bounding steps with a high knee lift, landing gently on the ball of your foot), plant & cut (Jog 4–5 steps, then plant on the outside leg and cut to change direction) (3 items, each item 2 sets)	

**Table 2:** The HarmoKnee training program, exercises and duration of the structured warm-up program applied

**Exercise**	**Duration**

**Warm-up**	**≥10 min**
Jogging (≥4–6 min), backward jogging on the toes (approximately 1 min), high-knee skipping (approximately 30 s), defensive pressure technique (approximately 30 s), one on one (≥2min) (encourage: straight alignment hip-knee-foot, low center of gravity, slightly flexed knees, soft and controlled landings)	
**Muscle activation**	**Approximately 2 min**
Activation of calf muscles, quadriceps muscles, hamstring muscles, hip flexor muscles, groin muscles, hip and lower back muscles (6 item, each item 4 s for each leg/side) (encourage: carefully hold and contract the muscle, focus on finding your muscle, stretch only in cases of a limited range of motion)	
**Balance**	**Approximately 2 min**
Forward and backward double leg jumps, Lateral single leg jumps, Forward and backward single leg jumps, Double leg jump with or without ball (optional), (4 items, each item approximately 30 s) (encourage: proper landing and takeoff in a jump, straight line hip-knee-foot with flexed knees, feet shoulder-width apart, soft and controlled landing, freeze the landing before taking off again, keep a low body-center of gravity, contract and hold stomach and buttocks during the whole exercise)	
**Strength**	**Approximately 4 min**
Walking lunges in place, hamstring curl (in pairs) (lay down on the ground, partner push your feet backward while you resist), single-knee squat with toes raised (3 items, each item approximately 1 min) (encourage: soft and controlled landing, contract stomach and buttocks, straight line hip-knee-foot)	
**Core stability**	**Approximately 3 min**
Sit-ups, plank on elbows and toes, bridging (lay on your back, keep your arm along the body and lift up your buttocks) (3 items, each item approximately 1 min) (encourage: contract stomach and buttocks, straight line through the body, breathe normally)	

**Table 3:** NPT of quadriceps in dominant and non-dominant leg (values are mean ± SD), and percentage of change (Δ) [values are mean (95% CI)] from the pre-test to post-test

	Dominant			Non-dominant		

NPT	Pre (Nm)	Post (Nm)	Δ% (95%CI)	Pre (Nm)	Post (Nm)	Δ% (95% CI)
**The 11+**						
Q30°	89.6±20.4	90.0±25.8	0.4(−12.5 to 13.3)	82.9±27.2	98.9±26.3	16.0(1.8 to 30.3)^*^
Q60°	179.7±37.2	198.8±47.7	19.1(1.7 to 36.4)^*^	169.9±40.1	205.3±53.8	35.3(13.9 to 56.7)^*^
Q90°	268.2±69.4	316.0±64.9	47.8(21.3 to 74.3)^*^	248.0±72.2	326.1±81.9	78.1(44.4 to 111.9)^*^
**HarmoKnee**						
Q30°	95.6±26.8	105.9±27.4	10.3(−11.3 to 31.9)	107.4±35.3	110.4±35.9	2.9(−22.6 to 28.6)
Q60°	186.1±49.3	228.2±56.4	42.2(−2.2 to 86.6)	207.1±53.6	228.5±62.9	21.4(−14.5 to 57.2)
Q90°	260.7±71.7	346.5±87.2	85.7(33.6 to 137.9)^*^	278.3±67.6	352.1±84.3	73.8(32.2 to 115.4)^*^
**Control**						
Q30°	97.3±24.8	97.6±23.0	0.3(−18.7 to 19.3)	99.3±25.2	85.2±19.2	−14.1(−39.0 to 10.8)
Q60°	208.6±31.9	184.2±34.3	−24.4(−57.1 to 8.3)	193.5±32.8	168.3±26.3	−25.1(−54.6 to 4.4)
Q90°	305.7±74.9	262.5±84.2	−43.1(−113.2 to 26.9)	271.6±55.5	241.2±80.1	−30.4(−86.6 to 25.7)

NPT= net peak torque; Q= Quadriceps muscles; pre= pre-test; post= post-test; Nm= Newton meter;

°= degree;

* p < 0.05.

**Table 4:** NPT of hamstrings muscle in dominant and non-dominant leg (values are mean ± SD), and percentage of change (Δ) [values are mean (95% CI)] from pre-test to post-test

	Dominant			Non-dominant		

NPT	pre (Nm)	post (Nm)	Δ% (95%CI)	pre (Nm)	post (Nm)	Δ% (95% CI)
**The 11+**						
H30°	142.6±37.8	167.5±31.5	24.8(7.1 to 42.6)^*^	121.3±34.9	150.0±28.2	28.7(13.0 to 44.3)^*^
H60°	114.2±26.2	134.1±25.9	19.8(7.4 to 32.3)^*^	101.7±28.5	115.4±22.1	13.7(4.9 to 22.4)^*^
H90°	90.3±25.3	100.5±17.9	10.2(−0.7 to 21.1)	86.0±19.1	90.6±14.5	4.5(−2.4 to 11.5)
**HarmoKnee**						
H30°	150.3±25.5	161.1±43.4	10.8(−15.3 to 37.0)	140.7±29.2	159.9±45.3	19.3(−5.5 to 44.1)
H60°	121.3±19.9	133.1±31.9	11.8(−11.4 to 34.9)	112.2±25.7	128.9±36.2	16.8(−7.5 to 41.1)
H90°	104.3±22.1	105.2±29.9	0.9(−19.7 to 21.6)	85.1±22.5	101.0±28.7	15.9(−1.5 to 33.3)
**Control**						
H30°	164.3±33.4	148.6±37.0	−15.6(−40.6 to 9.3)	143.2±31.3	128.7±28.1	−14.5(−37.2 to 8.2)
H60°	131.5±31.2	127.8±26.9	−3.8(−25.9 to 18.3)	122.1±25.3	111.8±22.1	−10.1(−24.3 to 3.9)
H90°	107.8±28.9	105.9±25.2	1.8(−19.9 to 23.6)	107.3±23.9	94.9±16.7	−12.4(−29.2 to 4.3)

NPT= net peak torque; H= Hamstring muscles; pre= pre-test; post= post-test; Nm= Newton meter;

°= degree;

* p<0.05.
